# *Patched1* haploinsufficiency severely impacts intermediary metabolism in the skin of *Ptch1*^+/−^/*ODC* transgenic mice

**DOI:** 10.1038/s41598-019-49470-w

**Published:** 2019-09-10

**Authors:** Changzhao Li, Bharat Mishra, Mahendra Kashyap, Zhiping Weng, Shaida A. Andrabi, Shahid M. Mukhtar, Arianna L. Kim, David R. Bickers, Levy Kopelovich, Mohammad Athar

**Affiliations:** 1 Department of Dermatology, University of Alabama at Birmingham, Birmingham, AL USA; 2 Department of Biology, University of Alabama at Birmingham, Birmingham, AL USA; 3 Department of Pharmacology and Toxicology, University of Alabama at Birmingham, Birmingham, AL USA; 4 Department of Neurology, University of Alabama at Birmingham, Birmingham, AL USA; 5 Department of Dermatology, Columbia University, New York, NY USA; 6 Department of Medicine, Weill Cornell Medical College, New York, NY USA

**Keywords:** Cancer metabolism, Basal cell carcinoma

## Abstract

The study of dominantly heritable cancers has provided insights about tumor development. Gorlin syndrome (GS) is an autosomal dominant disorder wherein affected individuals develop multiple basal cell carcinomas (BCCs) of the skin. We developed a murine model of Ptch1 haploinsufficiency on an ornithine decarboxylase (ODC) transgenic background (*Ptch1*^+/−^/ODC^t^/C57BL/6) that is more sensitive to BCCs growth as compared with *Ptch1*^+/+^/ODC^t^/C57BL/6 littermates. *Ptch1*^+/−^/ODC^t^/C57BL/6 mice show an altered metabolic landscape in the phenotypically normal skin, including restricted glucose availability, restricted ribose/deoxyribose flow and NADPH production, an accumulation of α-ketoglutarate, aconitate, and citrate that is associated with reversal of the tricarboxylic acid cycle, coupled with increased ketogenic/lipogenic activity via acetyl-CoA, 3-hydroybutyrate, and cholesterol metabolites. Also apparent was an increased content/acetylation of amino-acids, glutamine and glutamate, in particular. Accordingly, metabolic alterations due to a single copy loss of *Ptch1* in *Ptch1*^+/−^/ODC^t^/C57BL/6 heterozygous mice may provide insights about the cancer prone phenotype of BCCs in GS patients, including biomarkers/targets for early intervention.

## Introduction

Basal cell carcinomas (BCCs) of the skin represent nearly one-half of all cancers diagnosed in the United States^1,2^. Although majority of these tumors do not metastasize and death caused by these tumors is uncommon^3^, they are frequently associated with clinically significant morbidity caused by local invasion and tissue destruction. The basal cell nevus syndrome (BCNS), also known as Gorlin syndrome (GS) or the nevoid basal cell carcinoma syndrome (NBCCS), is an autosomal dominant disorder characterized by early onset of multiple BCCs, occasional childhood malignancies, and developmental defects^4,5^. The majority of GS cases are driven by mutations in the tumor suppressor gene (TSG) *Patched* (*Ptch*) and/or the G-protein coupled receptor smoothened (*SMO*)^6^, leading to activation of Gli transcription factors (Gli1, 2, 3), and thereby uncontrolled proliferation of basal cells and ultimately BCCs growth in the skin of affected individuals^7–10^.

In order to further define the pathogenesis of BCCs growth, we developed *Ptch1*^+/−^/ODC^t^/C57BL/6 transgenic mice wherein a single copy loss of *Ptch1* causes tumor initiation, and whereupon ornithine decarboxylase (ODC) transgene triggers tumor promotion^11,12^. Importantly, we have demonstrated that the histologic features of BCCs in *Ptch1*^+/−^/ODC^t^/C57BL/6 mice are similar to those in GS patients, including palisading at the edge of tumor nests, retraction from surrounding stroma, the lack of keratin pearl, and the characteristic expression pattern of marker biomolecules^11^.

Here, we show that *Ptch1* haploinsufficiency affects the metabolic landscape in phenotypically normal skin of *Ptch1*^+/−^/ODC^t^/C57BL/6 mice, wherein loss of a single copy of *Ptch1* in these mice caused a marked reduction of glucose utilization, coupled with reversal of the tricarboxylic acid (TCA) cycle through reductive carboxylation of α-ketoglutarate via glutamate^13,14^. This metabolic shift was associated with an accelerated metabolism of ketogenic/lipid metabolites through 3-hydroxybutyrate, and cholesterol derivatives. Incidentally, high lipid biosynthesis, in particular, was found to be associated with enhanced susceptibility to BCCs in GS patients^15^. Importantly, increased acetylation of specific amino acids/proteins, specifically increased content of glutamate/glutamine, and reduced cellular energy charge were demonstrated herein. Thus, targeting metabolites in the skin of *Ptch1*^+/−^/ODC^t^/C57BL/6 mice may enable potential interventional approaches of BCCs development^16–18^.

## Results

### *Ptch1* heterozygosity alters the metabolic landscape in phenotypically normal skin of *Ptch1*^+/−^/*ODC*^t^/C57BL/6 mice

**Figure 1:**
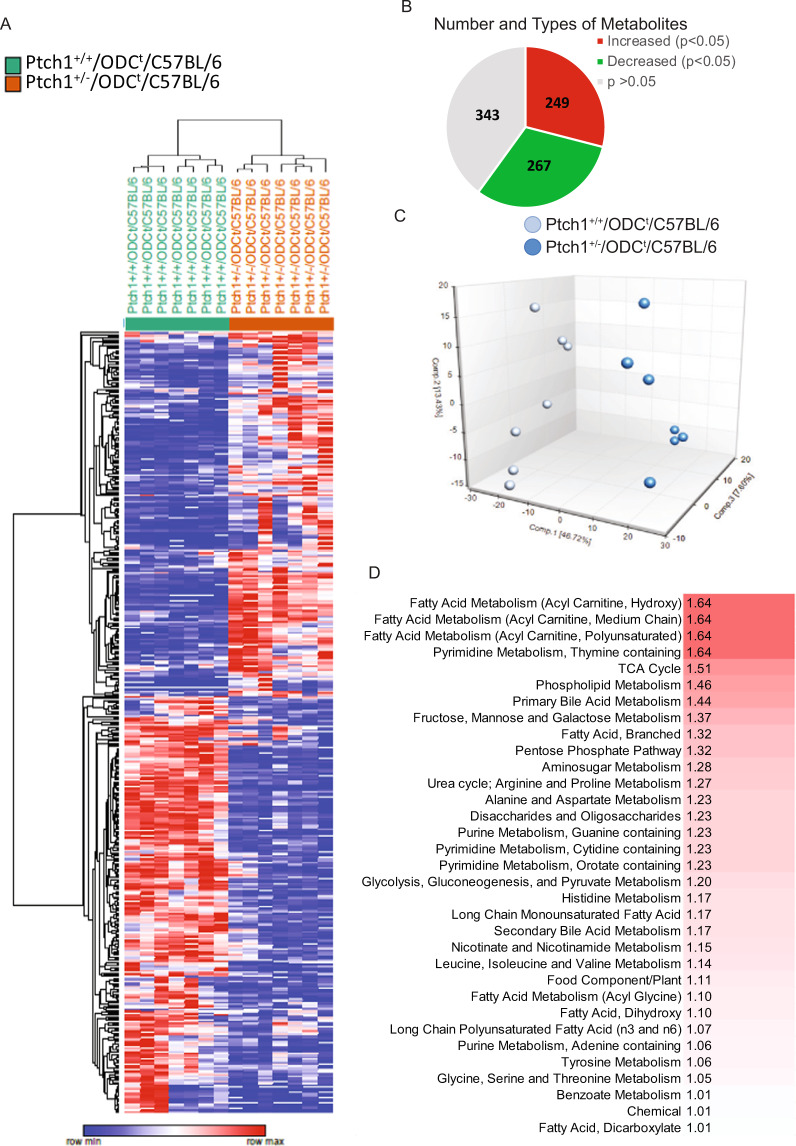
Effects of *Ptch1*^+/−^ heterozygosity on cutaneous metabolic landscape. (**A**) Clustering analysis of the abundance of named metabolites in the skin of *Ptch1*^+/+^/ODC^t^/C57BL/6 and *Ptch1*^+/−^/ODC^t^/C57BL/6 mice. Metabolites that were not significantly changed were not included in this analysis. Each row represents a single metabolite while each column represents an independent skin sample. The color scale indicates relative level of the abundance of each metabolite with red indicating levels greater than mean and blue indicating levels below the mean. (**B**) Pie chart showing numbers of biochemicals, which are increased or decreased in the skin; (**C**) Graph showing principle component analysis of global metabolomics data. (**D**) Graph showing the data of pathway set enrichment analysis (PSEA). Pathways containing greater than three identified metabolites are included in the analysis. The intensity of red color is associated with the PSEA score of each metabolite. PSEA score greater than one suggests the indicated pathway have above average number of metabolites that are significantly altered.

**Figure 2:**
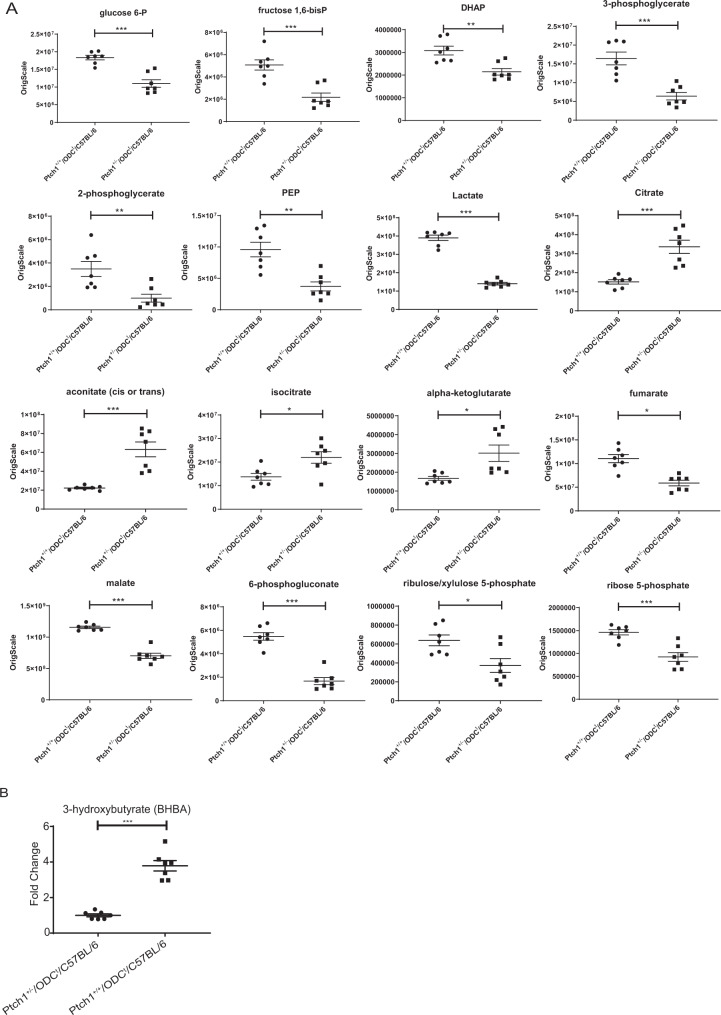
Effects of *Ptch1*^+/−^ heterozygosity on cutaneous glucose metabolism and pentose phosphate pathway. (**A**) Graphs showing the levels of various metabolites in glycolysis/TCA cycle/PPP; (**B**) Graph showing impact on ketone body, 3-hydroxybutyrate level. *p < 0.05; **p < 0.01; ***p < 0.001.

Metabolites identified by the “metabolon platform^TM^” in the phenotypically normal skin of *Ptch1*^+/+^/ODC^t^/C57BL/6 and *Ptch1*^+/−^/ODC^t^/C57BL/6 mice comprised a total of 859 biochemicals, 727 of which were of known identity (designated as “named” biochemicals) and 132 compounds were of unknown structural/functional identity (designated as “unnamed” biochemicals). Out of the 859 biochemicals, expression of 249 was increased while the expression of 267 was decreased significantly (p < 0.05). Out of the 132 unidentified metabolites, the expression of 37 was increased and expression of 24 was decreased significantly (p < 0.05) (Fig. 1A,B). Based on the metabolites’ pathway classification network by the “metabolon platform^TM^”, identified biochemicals were grouped into 8 recognized metabolic pathways (Supplementary Table 1). A principle component analysis (PCA) showed that the phenotypically normal skin specimens from *Ptch1*^+/−^/ODC^t^/C57BL/6 mice were clearly distinguished from their *Ptch1*^+/+^/ODC^t^/C57BL/6 littermates, indicating that introduction of *Ptch1* heterozygosity significantly alters the skin metabolome of these mice (Fig. 1C). A PCA that did not include “unnamed” metabolites has shown essentially a similar pattern (not shown). The metabolic profile of the major pathways was as follows:

In the carbohydrate metabolic pathway, 7 biochemicals were found to be significantly elevated in *Ptch1*^+/−^/ODC^t^/C57BL/6 mice, including lactose, raffinose, sucrose, ribitol, galactitol, and fucose (Supplementary Table 1). Yet, the top 10 metabolites that were found to be significantly decreased were 2-phosphglycerate, 6-phosphogluconate, N-acetylglucosamine 6-phosphate, galactonate, lactate, phosphoenolpyruvate (PEP), 3-phosphoglycerate, fructose 1,6-diphosphate/glucose 1,6-diphosphate/myo-inositol diphosphates, sedoheptulose, and glucuronate (Supplementary Table 1). The majority of these biochemicals represent key intermediates of the glycolytic and pentose phosphate pathways (PPP), suggesting a major reduction in cellular functions that regulate glucose utilization through glycolysis, including reduced ribose/deoxyribose for nucleic acids biosynthesis, as well as NADPH levels that is largely produced through the PPP. NADPH is a major contributor for fatty acid bio-synthesis^19^. Significantly, tightly linked downstream metabolites which comprise the “entry/exist” portion of the TCA cycle, including citrate, aconitate and α-ketoglutarate were considerably elevated (Fig. 2A), while other downstream TCA cycle intermediates were significantly reduced.

The 10 most elevated biochemicals in the lipid class were associated with bile acids and steroid metabolism, including taurodeoxycholate, tauro-beta-muricholate, taurocholate, taurochenodeoxycholate, tauroursodeoxycholate, taurohyodeoxycholic acid, chenodeoxycholate, 18-methylnonadecanoate (i20:0), corticosterone and dicarboxylate fatty acid sebacate which levels were increased between 3.9 to 130.9 -fold in the skin of *Ptch1*^+/−^/ODC^t^/C57BL/6 mice, as compared to *Ptch1*^+/+^/ODC^t^/C57BL/6 mice (Supplementary Table 1). The 10 most diminished biochemicals in the lipid class were linolenoylcarnitine (C18:3), 1-palmitoyl-2-oleoyl-GPG (16:0/18:1), decanoylcarnitine (C10), octanoylcarnitine (C8), glycerophosphoethanolamine, (S)-3-hydroxybutyrylcarnitine, (R)-3-hydroxybutyrylcarnitine, mead acid (20:3n9), N-palmitoleoyltaurine, and eicosapentaenoate (EPA; 20:5n3) which levels were decreased between 0.15 to 0.36- fold relative to their levels in *Ptch1*^+/+^/ODC^t^/C57BL/6 mice (Supplementary Table 1). Interestingly, the 10 most diminished lipid metabolites were essentially all carnitine derivatives that are associated with fatty acids transport across the mitochondrial membrane for energy utilization, including phospholipids^20^. The decrease in fatty acids and complex phospholipids is presumably due to the sharp decrease of 3-carbon intermediates (glycerol based) in the glycolytic pathway that, in general, provide the backbone for the synthesis of these lipids. These results reinforce the selective utility of acetyl CoA in the augmentation of 3-hydroxybutyrate and cholesterol metabolites, but not fatty acids or complex phospholipids.

Changes in the amino acids metabolic pathways included the top 10 increased metabolites, comprising N2,N6-diacetyllysine, phenol glucuronide, N-acetylcitrulline, N-acetylglutamine, formiminoglutamate, phenol sulfate, p-cresol glucuronide, N2, N5-diacetylornithine, 4-hydroxyphenylacetate sulfate, and N-acetylglutamate which were increased between 6.5 to 34.2 fold in *Ptch1*^+/−^/ODC^t^/C57BL/6 mice as compared to their levels in Ptch1^+/+^/ODC^t^/C57BL/6 littermates (Supplementary Table 1). The amino acid, N2, N6-diacetyllysine was increased 34.2- fold. In addition, 4 out of these 10 metabolites were enriched in the tyrosine metabolic pathway, including phenol glucuronide, phenol sulfate, p-cresol glucuronide, and 4-hydroxyphenylacetate sulfate. The other 5 metabolites are known to be important intermediates of the urea cycle, including arginine and proline metabolites: N-acetylcitrulline, and N2,N5-diacetylornithine; glutamate metabolites: N-acetylglutamine, N-acetylglutamate, formiminoglutamate, and histidine metabolites (Supplementary Table 1). In this regard, the enrichment of glutamine/glutamate metabolites, in particular, is highly relevant to the reversal of the TCA cycle via reductive carboxylation of α-ketoglutarate^13,14^. Also intriguing, are the high levels we found of n-acetyl-lysines which might reflect the increased content of the acetyl-CoA pool as well as their potential role as potent epigenetic regulators^21^.

In the nucleotides metabolism super pathway, the 10 most elevated metabolites (between 2.02 fold to 7.85 fold) were the following purines and pyrimidines 5,6-dihydrothymine, adenosine-2′3′-cyclic monophosphate, N-carbamoylaspartate, guanosine-2′3′-cyclic monophosphate, adenine, orotate, dihydroorotate, 5,6-dihydrouracil, cytosine, and 1-methylhypoxanthine whereas the 10 most decreased metabolites (between 0.13 to 0.55 fold) were N6-succinyladenosine, uric acid ribonucleoside, N1-methyladenosine, thymidine 3′-monophosphate, 2′-deoxycytidine 5′-monophosphate, N1-emthylguanosine, 2′-deoxyadenosine 5′-monophosphate, 2′deoxyadenosine, orotidine, 2′-deoxyguanosine 5′-monophosphate (dGMP) (Supplementary Table 1). It is interesting to note that orotate has been shown as a tumor promoter in hepatocarcinogenesis^22^.

In the vitamin and cofactors metabolism pathway, 4 biochemicals including nicotinamide riboside, nicotinate, 1-methylnicotinamide, and quinolinate are found to be increased between 2.05 to 5.77 fold relative to *Ptch1*^+/+^/ODC^t^/C57BL/6 littermates (Supplementary Table 1). Interestingly, these are all enriched in nicotinate and nicotinamide metabolism. On the other hand, 7 biochemicals which include dehydroascorbate, pyridoxate, N1-methyl-4-pyridone-3-carboxamide, biliverdin, N1-methyl-2-pyridone-5-carboxamide, oxalate, and pantothenate are decreased between 0.27 to 0.75 fold as compared to *Ptch1*^+/+^/ODC^t^/C57BL/6 littermates (Supplementary Table 1).

The top10 increased xenobiotics were 3-formylindole, formononetin, indolin-2-one, biochanin A, p-cresol sulfate, soyasaponin II, soyasaponin I, indole-3-acetamide, and soyasaponin III which were increased between 4.0 to 22.2 fold as compared to their levels in *Ptch1*^+/+^/ODC^t^/C57BL/6 (Fig. 1A & Supplementary Table 1). The top 10 diminished xenobiotics were 4-ethylphnylsuflate, ferulic acid 4-sulfate, 3-(3-hydroxyphenyl) propionate sulfate, N-acetylpyrraline, 2-hydroxyhippurate, 1,2,3-benzenetriol sulfate (2), catechol sulfate (Supplementary Table 1).

The Online Metabolon software^TM^ Pathway Set Enrichment Analysis (PSEA), showed that a majority of significantly modulated pathways comprised carbohydrate/TCA cycle, lipids, amino acids, and nucleotide metabolism (Fig. 1D & Supplementary Table 2).

### *Ptch1* heterozygosity alters cutaneous energy metabolism

Increased glucose utilization via glycolysis is prevalent in cancer cells^16^. Here, however, we found that *Ptch1* heterozygosity in *Ptch1*^+/−^/ODC^t^/C57BL/6 mice suppressed glucose utilization as is evidenced by a significant decrease in multiple intermediates of the glycolytic cascade (Fig. 2A,B), including a profound decrease of more than 50% in the levels of fructose 1,6-diphosphate, dihydroxyacetone phosphate (DHAP), 3-phosphoglycerate, 2-phosphoglycerate, phosphoenolpyruvate, and lactate as compared to their *Ptch1*^+/+^/ODC^t^/C57BL/6 littermates (Fig. 2A). As for the TCA cycle, while citrate, cis-aconitate, and α-ketoglutarate were increased significantly (Fig. 2A), there was a significant decrease in the level of fumarate (48%) and malate (40%). This metabolic pattern is remarkable, suggesting reversal of the TCA cycle due to reductive carboxylation of α-ketoglutarate via glutamate^13,14^, leading back through citrate to increased acetyl-CoA by the citrate cleavage enzyme (ATP citrate lyase)^23^ (Fig. 2A). Thus, *Ptch1* heterozygosity alters skin energy metabolism, potentially leading to a metabolic state that appears to prefer production of acetyl-CoA from TCA cycle reversal, including presumably a limited production of acetyl-CoA through fatty acid β-oxidation^24^. The latter is consistent with a significant increase in 3-hydroxybutyrate (Fig. 2B), supporting a switch towards ketone body-dependent energy metabolism. It is also of interest that 2-methylcitrate was significantly enhanced. This metabolite was shown to alter mitochondrial membrane permeability pore transition^25^.

### *Ptch1* heterozygosity suppressed cutaneous pentose phosphate pathway (PPP) in *Ptch1*^+/−^/ODC^t^/C57BL/6 mice

Since we observed decreased glycolysis in *Ptch1* heterozygous mice, such as reduction of about 40% in glucose 6-phosphate, we further assessed the impact of *Ptch1* gene dose on the PPP. This pathway involves a irreversible conversion of glucose 6-phosphate to ribulose 5-phosphate, wherein the latter was found to be significantly decreased. Moreover, all other intermediates of this pathway including 6-phosphogluconate, ribulose 5-phosphate, xylulose 5-phosphate, and ribose 5-phosphate were significantly reduced. Among these, 6-phosphogluconate was decreased by 72% (Fig. 2A). Obviously, a severe reduction of the PPP would, in turn, affect ribose/deoxyribose levels, and thereby nucleosides/nucleotides for energy production and DNA synthesis. Furthermore, the PPP also serves as a major resource for the production of NADPH that is largely used for de-novo fatty acids synthesis.

### *Ptch1* heterozygosity alters cutaneous lipid metabolism in *Ptch1*^+/−^/ODC^t^/C57BL/6 mice

**Figure 3:**
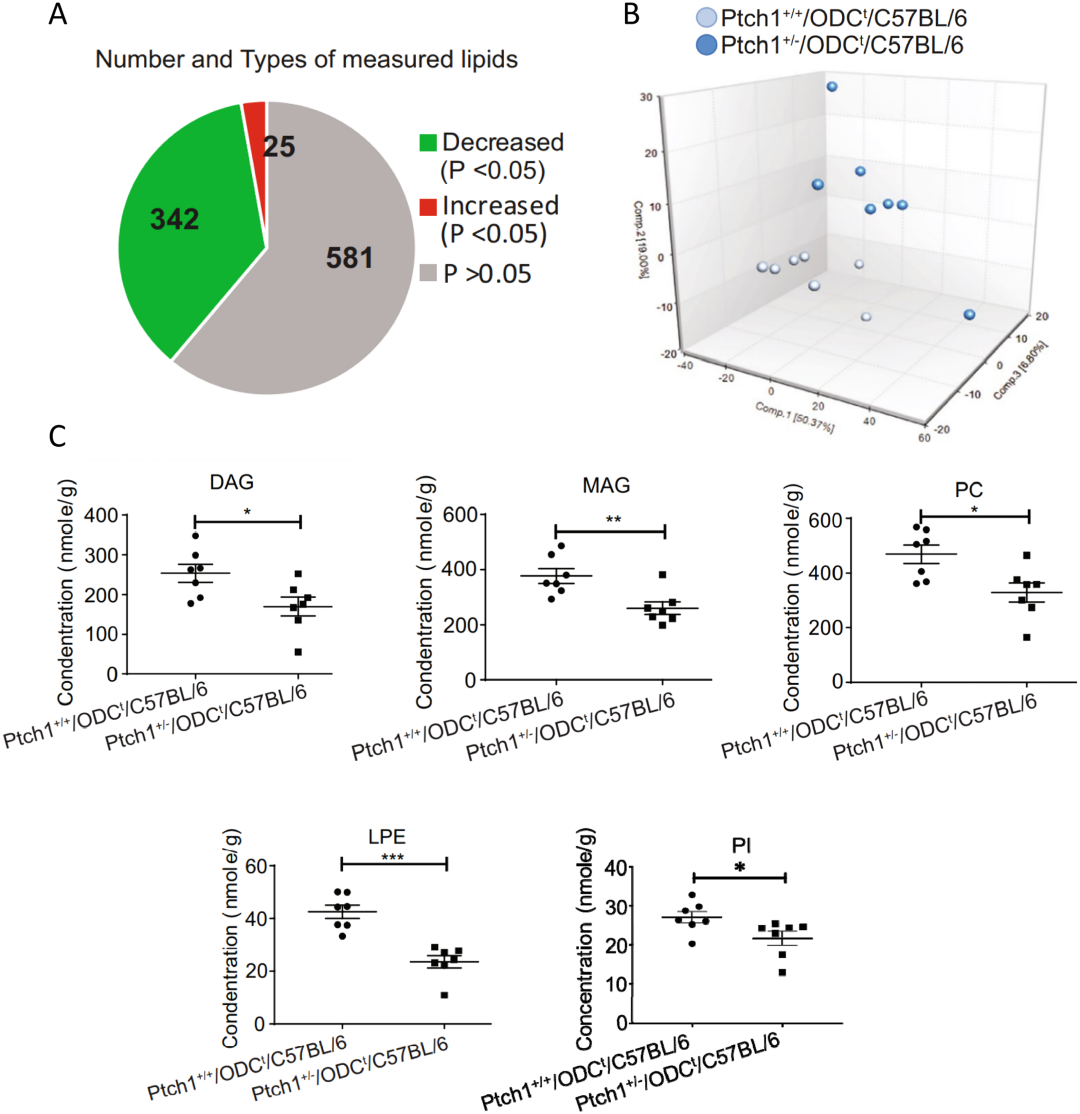
Effects of Ptch1^+/−^ heterozygosity on lipid profile in the skin. (**A**) Pie chart depicting the numbers of lipids, which are increased or decreased in the skin; (**B**) Graph showing principle component analysis of lipidomics data; (**C**) Graphs showing impact on mono & diacylglycerols, phosphatidylcholines, lysophosphatidylethanolamines, and phosphatidylinositol. *p < 0.05; **p < 0.01; ***p < 0.001.

In the lipidomics profiling we employed a “complex lipids platform” in order to gain further insight into the effects of *Ptch1* heterozygosity on cutaneous lipid metabolism. As illustrated in Fig. 3A, out of 948 identified biochemical lipid metabolites, only 25 were found to be increased, 342 decreased and the remaining 581 showed no significant changes (Supplementary Table 3). A PCA of the complex lipids data suggest intra-group variations among the *Ptch1*^+/−^/ODC^t^/C57BL6 specimens, which, however, were separated from of *Ptch1*^+/+^/ODC^t^/C57BL/6 littermates specimens, falling into two distinct clusters (Fig. 3B). The decreased lipids consisted primarily of fatty acids, phospholipids as well as diacylglycerols (DAG) and monoacylglycerols (MAG), with no significant changes in sphingolipids (Fig. 3C & Supplementary Table 4). Yet, phosphatidylcholines (PC), phosphatidylinositols (PI) and lysophophatidylethanolamines (LPE) represented the three major groups showing significant reduction in this category of phospholipid (Fig. 3C & Supplementary Table 4). The decrease of complex phospholipids may be due to a severe reduction of 3-carbon intermediates via the glycolytic pathway^26^ (Fig. 2A).

### *Ptch1* heterozygosity promotes cutaneous cholesterol metabolism in *Ptch1*^+/−^/ODC^t^/C57BL/6 mice

**Figure 4:**
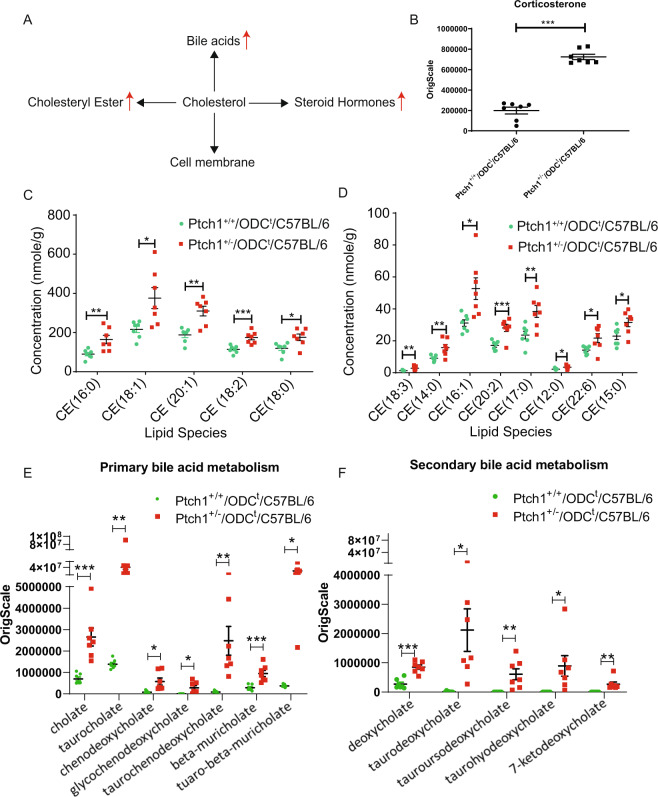
Effects of *Ptch1*^+/−^ heterozygosity on cholesterol metabolism. (**A**) Flow diagram of cholesterol dependent metabolic pathways in skin; (**B**–**F**) Graphs showing the impact of *Ptch1*^+/−^ heterozygosity on corticosterone (**B**), cholesteryl esters (**C,D**), primary bile acid metabolites  (**E**) and secondary bile acid metabolites (**F**). *p < 0.05; **p < 0.01; ***p < 0.001.

Interestingly, the only lipid group which showed higher cutaneous accumulation is cholesteryl esters (CEs) (p = 0.0578) (Supplementary Table 3). This may be an expected finding confirming the role of *PTCH1* protein in cholesterol transport^8^. Cholesterol metabolism is associated with the biosynthesis of corticosteroids, bile acids, vitamin D, and cell membrane components (Fig. 4A)^27–29^. As listed in Fig. 4C,D & Supplementary Table 5, various types of Cholesterol esters (CEs) were significantly upregulated in the phenotypically normal skin of *Ptch1*^+/−^/ODC^t^/C57BL/6 mice. Other important cholesterol metabolites are glucocorticoids (such as cortisol in humans and corticosterone in rodents). A 4.2-fold increase of corticosterone was noted in phenotypically normal skin of *Ptch1*^+/−^/ODC^t^/C57BL/6 as compared to *Ptch1*^+/+^/ODC^t^/C57BL/6 (Fig. 4B), suggesting a potential role in inflammation during the process of BCCs development^30^.

Further analysis showed that primary and secondary bile acid metabolites appear to be more significantly upregulated; between 3.3 to 130.9 - fold (Fig. 4E,F). The most upregulated metabolite in bile acid metabolism was taurodeoxycholate of 130- fold, followed by tauro-beta-muricholate of 42.0- fold (Fig. 4E,F & Supplementary Table 1). Taurodeoxycholate has been demonstrated to increase proliferation and to inhibit apoptotic cell death through activation of NF-kB^31^. Other biological active bile acid metabolites were also highly upregulated, including taurocholate (23.3- fold), taurochenodeoxycholate (19.8- fold), tauroursodeoxycholate (9.5- fold) and taurohyodeoxycholate (8.5- fold) (Fig. 4E,F & Supplementary Table 1). It is important to note that bile acids are physiological ligands of farnesoid X receptor (FXR). FXR regulates the expression of genes controlling cholesterol homeostasis, including other lipids, and glucose metabolism which were shown to positively affect tumorigenesis^32,33^. Incidently, heteromeric complexes of FXR with RXR play an important role in retinoic acid metabolism, including its clinical application to control inflammation and certain cancers^34,35^.

### Cutaneous alterations of nucleic acid metabolism by *Ptch1* heterozygosity in *Ptch1*^+/−^/ODC^t^/C57BL/6 mice

ODC provides a pool of polyamines which are precursors of purines and pyrimidines^36^. Significantly, adenosine 2′,3′-monophosphate (cAMP) and guanosine 2′,3′-monophosphate (cGMP) were consistently elevated by 5.1 and 4.4- fold, respectively. Similarly, the levels of purine containing nucleotide/nucleosides catabolites such as inosine, guanosine and hypoxanthine were higher in *Ptch1*^+/−^/ODC^t^/C57BL/6 than in their control littermates, albeit these did not achieve statistical significance (Table 1). Additionally, guanine and adenine were significantly increased by 1.6 and 3.9- fold, respectively (Table 1). Interestingly, the level of both 2′-deoxy adenosine 5′-monophosphate (dAMP) and 2′-deoxy guanosine 5′-monophosphate (dGMP) were decreased, including adenine and guanine containing nucleosides, suggesting that shortage of deoxyribose due to severe reduction of the PPP (vide supra) might be implicated. These examples include N1-methyladenosine, N6-carbamoylthreonyladenosine, N6-succinyladenosine, and N1-methylguanosine. Urate, the end product of purine catabolism, was not substantially altered, but its metabolite allantoin was found to be increased significantly in the skin of *Ptch1*^+/−^/ODC^t^/C57BL/6 mice (Table 1).

**Table 1:** Effects of Ptch1 heterozygosity on nucleotide metabolism.

Biochemical	Fold change	p value
**Purine Metabolism**		
inosine	1.93	0.0347
hypoxanthine	1.65	0.0238
N1-methylinosine	0.68	0.0003
urate	0.84	0.0090
uric acid ribonucleoside*	0.41	0.0000
allantoin	1.8	0.0003
2′-AMP	1.6	0.0185
adenosine-2′,3′-cyclic monophosphate	5.12	0.0004
adenine	3.92	0.0045
1-methyladenosine	0.42	0.0000
N6,N6-dimethyladenosine	1.35	0.0093
N6-carbamoylthreonyladenosine	0.59	0.0000
2′-deoxyadenosine 5′-monophosphate	0.49	0.0000
2′-deoxyadenosine	0.52	0.0036
N6-succinyladenosine	0.13	0.0000
guanosine 3′-monophosphate (3′-GMP)	1.32	0.0153
guanosine-2′,3′-cyclic monophosphate	4.38	0.0055
guanosine	1.78	0.0190
7-methylguanine	0.69	0.0000
2′-O-methylguanosine	1.21	0.0029
1-methylguanosine	0.45	0.0000
N2,N2-dimethylguanosine	0.57	0.0000
2′-deoxyguanosine 5′-monophosphate (dGMP)	0.55	0.0002
guanosine 2′-monophosphate (2′-GMP)*	1.72	0.0077
**Pyrimidine Metabolism**		
dihydroorotate	3.37	0.0227
orotate	3.42	0.0005
orotidine	0.55	0.0000
**Pyrimidine Metabolism, Uracil containing**		
uridine-2′,3′-cyclic monophosphate	1.57	0.0050
uridine	0.73	0.0003
uracil	0.68	0.0000
5,6-dihydrouridine	0.68	0.0000
5-methyluridine (ribothymidine)	0.7	0.0004
5,6-dihydrouracil	3.03	0.0000
2′-deoxyuridine	0.59	0.0000
uridine 2′-monophosphate (2′-UMP)*	1.4	0.0004
3-(3-amino-3-carboxypropyl)uridine*	0.63	0.0000
cytidine 2′,3′-cyclic monophosphate	1.56	0.0093
cytidine	0.81	0.0464
cytosine	2.74	0.0014
3-methylcytidine	0.84	0.0130
5-methylcytidine	0.59	0.0000
N4-acetylcytidine	0.73	0.0011
dCMP	0.44	0.0001
2′-deoxycytidine	0.81	0.0015
5-methyl-2′-deoxycytidine	0.61	0.0009
TMP	0.59	0.0000
thymidine 3′-monophosphate	0.43	0.0003
thymidine	0.73	0.0073
thymine	0.8	0.0205
5,6-dihydrothymine	7.85	0.0000
methylphosphate	0.63	0.0000

Pyrimidine metabolism, on the other hand, was significantly augmented. The precursor metabolites of the pyrimidine *de novo* synthetic pathway, including N-carbamoylaspartate, dihydroorotate, and orotate were increased significantly by 4.9, 3.4 and 3.4-fold respectively (Table 1). The levels of uridine 5′-monophosphate (UMP), which is converted to cytidine or thymidine containing nucleotides for DNA synthesis, did not change appreciably, yet both cyclic UMP (cUMP) and cyclic CMP (cCMP) were increased significantly. The latter two metabolites have recently been demonstrated as second messenger and they play essential role in cell proliferation^37^. In contrast, deoxynucleic acid thymidine 5′-monophosphate (TMP) and 2′-deoxycytidine 5′-monophosphate (dCMP) were decreased substantially by 41% and 56% respectively, consistent with increased accumulation of related catabolites including cytosine, dihydrouracil and dihydrothymine (2.7, 3.0 and 7.9- fold respectively) (Table 1).

## Discussion

Identification of TSGs and their effector pathways in several autosomal dominant hereditary cancer syndromes has provided insights about the underlying mechanisms of cancer initiation and potential therapeutic intervention^38–40^. GS is a dominantly heritable cancer syndrome in which BCCs arise through a two-hit mechanism wherein “one hit” is an inherited, inactivating mutation in *PTCH1*, and the second hit is a somatically derived mutation in the remaining *PTCH1* allele^41^. In sporadic BCCs, the majority of the cases harbor two somatic mutations in *PTCH1*, or less often, an activating mutation in *SMO* mimicking loss of *PTCH1*^10,41–45^, although additional molecular changes are probably necessary for BCCs development.

In order to model the state of *PTCH1* haploinsufficiency in GS patients, the present study focused on the global expression profile of metabolites in the phenotypically normal skin of *Ptch1*^+/−^/ODC^t^/C57BL/6 mice and their *Ptch1*^+/+^/ODC^t^/C57BL/6 littermates, leading to the following important findings.

First, we found a sharp decrease of key glycolytic intermediates in the skin of *Ptch1*^+/−^/ODC^t^/C57BL/6 mice, severely curtailing glucose flow/utilization and ATP production therefrom.

Second, we found a sharp decrease of key intermediates of the PPP in the skin of *Ptch1*^+/−^/ODC^t^/C57BL/6 mice, affecting the flow of ribose/deoxyribose for nucleosides/nucleotides synthesis, as well as production of reduced NADP that is a major resource for de-novo fatty acids synthesis.

Third, we identified a metabolic sequence that is associated with reversal of the TCA cycle through reductive carboxylation of α-ketoglutarate via glutamate, leading to an accumulation of citrate, and thereby an abundance of acetyl CoA in the skin of *Ptch1*^+/−^/ODC^t^/C57BL/6 mice. Notably, reversal of the TCA cycle would largely curtail ATP production, as well.

Fourth, in support of these results we found a significant increase in the level of 3-hydroxybutyric acid, a ketone body, which is formed from acetyl-CoA through acetoacetate, and is used as an energy source when glucose supply is low or absent and when reversal of the TCA cycle occurs. Apparently, increased levels of 3-hydroxybutyrate as a major source of energy is associated with an activated HH pathway in the skin of *Ptch1*^+/−^/ODC^t^/C57BL/6 mice. Incidentally, 3-hydroxybutyric acid levels were also found to promote tumorigenesis by modulating H3K9 acetylation^46^.

Fifth, we found a significant increase of cholesterol metabolites, including taurine esters of bile acids. This reflect, in part, the utility of acetyl CoA under conditions of low glucose where, in addition to 3-hdroxybutyrate for energy, the cholesterol biosynthetic pathway is selectively activated in the skin of *Ptch1*^+/−^/ODC^t^/C57BL/6 mice. The fact that other lipid forms were not affected apparently due to severe reduction of glycolytic intermediates, specifically glycerol containing moieties, which are the prime source for the synthesis of complex phospholipids. Bile acids are known modulators of FXR signaling and they could play an important role in maintaining the high propensity for BCCs induction that is associated with GS^47^.

Sixth, the changes we observed in amino acids metabolites may reflect the increased acetylation pool that would affect the regulatory potential of proteins as well as the role of acetylated individual amino acids, including their metabolic interconversion pathways^48^ during the early development of BCCs. Of particular interest is the apparent abundance of glutamine/glutamate (both acetylated and unacetylated) as compared with α-ketoglutarate levels (about 16 fold) which is a key causal factor to affect reversal of the TCA cycle in the skin of *Ptch1*^+/−^/ODC^t^/C57BL/6 mice. Another important consideration is based on evidence that reversal of the TCA cycle via glutamate dehydrogenase (GDH) is specifically associated with a low cellular energy charge, i.e., decreased ATP levels^49^. Indeed, the general pattern that emerges in the present study regarding alterations that involve the purine/pyrimidine pools is one of reduced synthesis of di-tri-phosphonucleotides.

**Figure 5:**
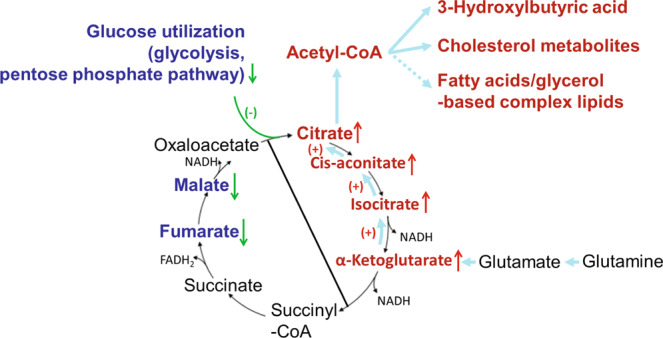
Metabolic alterations in the skin during the initiation phase of BCCs development in the skin of *Ptch1*^+/−^/ODC^t^/C57BL/6 mice. Detailed description of this model has been included in discussion.

In summary, our experimental model (Fig. 5) specifies that metabolic profiling of the skin in *Ptch1*^+/−^/ODC^t^/C57BL6 mice during the initiation phase of BCCs development^38^ can identify novel molecular biomarkers/targets for chemoprevention of BCCs risk. Most importantly is apparently the glutamine pathway^50^ that under conditions of glucose shortage plays a key role in the overall metabolic pattern that emerges herein, including reduced glucose utilization, coupled with reversal of the TCA cycle, limited cellular energy charge, as well as increased reliance on a specific subset of lipid metabolism, i.e., 3-hydroxybutyrate and cholesterol metabolites, including bile acids. Specifically, therefore, relevant biomarkers/targets would presumably consist of the urea/ammonia cycle *vis*. glutamine synthesis^50^, followed by glutamine/glutamate contribution to reductive carboxylation of a-ketoglutarate^14^, citrate metabolism *vis*. ATP citrate lyase (citrate cleavage enzyme)^23^, and HMG-CoA reductase that is integral to the synthesis of cholesterol metabolites^7,8^. Whether some or all of these sites might be due to *Ptch1* haploinsufficiency or an activating mutation in SMO, remains to be established. We have previously shown that cyclopamine, and perhaps other SMO antagonists, are potent *in vivo* inhibitors of UVB-induced BCC in *Ptch1*^+/−^ mice and most likely in humans^51^.

## Materials and Methods

### Animals

We developed *Ptch1*^+/−^/ODC^t^/C57BL/6 murine model in which *Ptch1* mutations drive aberrant activation of Sonic hedgehog (SHH) signaling^11^. These mice were generated by deletion of exons 1 and 2 of the *Ptch1* gene. ODC transgene overexpression in the skin of these mice was driven by a K6 promoter near the hair follicles where basal skin cells presumably reside^52^. The male *ODC*^*t*^ breeders (6–7 weeks old) were purchased from Taconic (Germantown, NY, USA). The detailed breeding protocols and genotyping of *Ptch1*^+/−^/ODC^t^/C57BL/6 animals are described earlier^11^. Of note, none of the animals, whether *Ptch1* wild type or *Ptch1* mutated, studied herein were exposed to UVB or other pro-carcinogenic environmental stimuli. Phenotypically normal skin is defined here as untreated skin of animals with no visible neoplastic lesions. Hence, no difference in skin phenotype was observed between *Ptch1*^+/−^/ODC^t^/C57BL/6 and *Ptch1*^+/+^/ODC^t^/C57BL/6 mice. All animal care and experimental protocols were approved by the Institutional Animal Care and Use Committee (IACUC) at the University of Alabama at Birmingham and Columbia University. All experiments were carried out in accordance with relevant guidelines and regulations.

### Skin tissue samples preparation

After the termination of the experiment, skin tissues from each animal were harvested, snap frozen and stored in −80 °C freezer. At least 100 mg of skin tissue from each animal was used for the metabolon and lipid analysis. At least eight skin samples from eight animals were analyzed in each group.

### Metabolon platform

Sample preparation, data collection and data analysis were performed by standard protocol of Metabolon (Morrisville, NC, USA). Briefly, samples were prepared using the automated MicroLab STAR® system from Hamilton Company. Proteins were precipitated with methanol under vigorous shaking for 2 min (Glen Mills GenoGrinder 2000) followed by centrifugation. The resulting extract was divided into five fractions: two for analysis by two separate reverse phase (RP)/UPLC-MS/MS methods with positive ion mode electrospray ionization (ESI), one for analysis by RP/UPLC-MS/MS with negative ion mode ESI, one for analysis by HILIC/UPLC-MS/MS with negative ion mode ESI, and one sample was reserved for backup. Samples were placed briefly on a TurboVap® (Zymark) to remove the organic solvent. The sample extracts were stored overnight under nitrogen before preparation for analysis. Several types of controls and standard were analyzed in concert with the experimental samples to ensure the data quality. The sample extracts were analyzed by ultrahigh performance liquid chromatography-tandem mass spectroscopy (UPLC-MS/MS) followed by data extraction and compound identification, curation, metabolite quantification and data normalization. Detailed methods can be found in Supplementary Materials^53^.

### Complex lipid platform

Lipids were extracted from samples in methanol:dichloromethane in the presence of internal standards. The extracts were concentrated under nitrogen and reconstituted in 0.25 mL of 10 mM ammonium acetate dichloromethane:methanol (50:50). The extracts were transferred to inserts and placed in vials for infusion-MS analysis, performed on a Shimazdu LC with nano PEEK tubing and the Sciex SelexIon-5500 QTRAP. The samples were analyzed via both positive and negative mode electrospray. The 5500 QTRAP scan was performed in MRM mode with the total of more than 1,100 MRMs. Individual lipid species were quantified by taking the peak area ratios of target compounds and their assigned internal standards, then multiplying by the concentration of internal standard added to the sample. Lipid class concentrations were calculated from the sum of all molecular species within a class, and fatty acid compositions were determined by calculating the proportion of each class comprised by individual fatty acids^54^.

### Pathway set enrichment and principle component analyses

Pathway set enrichment analysis (PSEA) was performed by Metabolync portal: https://retiredportal.metabolon.com. The pathway enrichment value is calculated by comparing the ratio of significantly changed compounds in a particular pathway to the ratio of significantly altered compounds relative to all named compounds in the study.

PCA is an unsupervised analysis that reduces the dimension of the data, and where the total variance is defined as the sum of the variances of the predicted values of each component and for each component, the proportion of the total variance was computed by Metabolon (Morrisville, NC, USA).

We should note that the Metabolon platform used herein cannot detect “high-energy” compounds, nor can it detect volatile metabolites, some of which are considered in the context of this study; for example, acetyl-CoA, di-and tri-phosphonucleotides, acetate, and oxaloacetate. However, these are being inferred based on the current understanding of metabolomics.

### Statistical analysis

Student’s *t*-test was used to compare the mean of the two groups. In all calculations, p < 0.05 was considered statistically significant.

## Supplementary information

Supplementary Dataset 1

Supplementary Dataset 2

Supplementary Dataset 3

Supplementary Dataset 4

Supplementary Dataset 5
