# Author Correction: *Patched1* haploinsufficiency severely impacts intermediary metabolism in the skin of *Ptch1*^+/−^/*ODC* transgenic mice

**DOI:** 10.1038/s41598-021-98190-7

**Published:** 2021-10-11

**Authors:** Changzhao Li, Bharat Mishra, Mahendra Kashyap, Zhiping Weng, Shaida A. Andrabi, Shahid M. Mukhtar, Arianna L. Kim, David R. Bickers, Levy Kopelovich, Mohammad Athar

**Affiliations:** 1 Department of Dermatology, University of Alabama at Birmingham, Birmingham, AL USA; 2 Department of Biology, University of Alabama at Birmingham, Birmingham, AL USA; 3 Department of Pharmacology and Toxicology, University of Alabama at Birmingham, Birmingham, AL USA; 4 Department of Neurology, University of Alabama at Birmingham, Birmingham, AL USA; 5 Department of Dermatology, Columbia University, New York, NY USA; 6 Department of Medicine, Weill Cornell Medical College, New York, NY USA

Correction to: *Scientific Reports* 10.1038/s41598-019-49470-w, published online 10 September 2019

The original version of this Article contained errors in Figure 3C, where the graph showing “PI” was a duplication of “LPE”.

The original Figure 3 and accompanying legend appear below.

**Figure 3:**
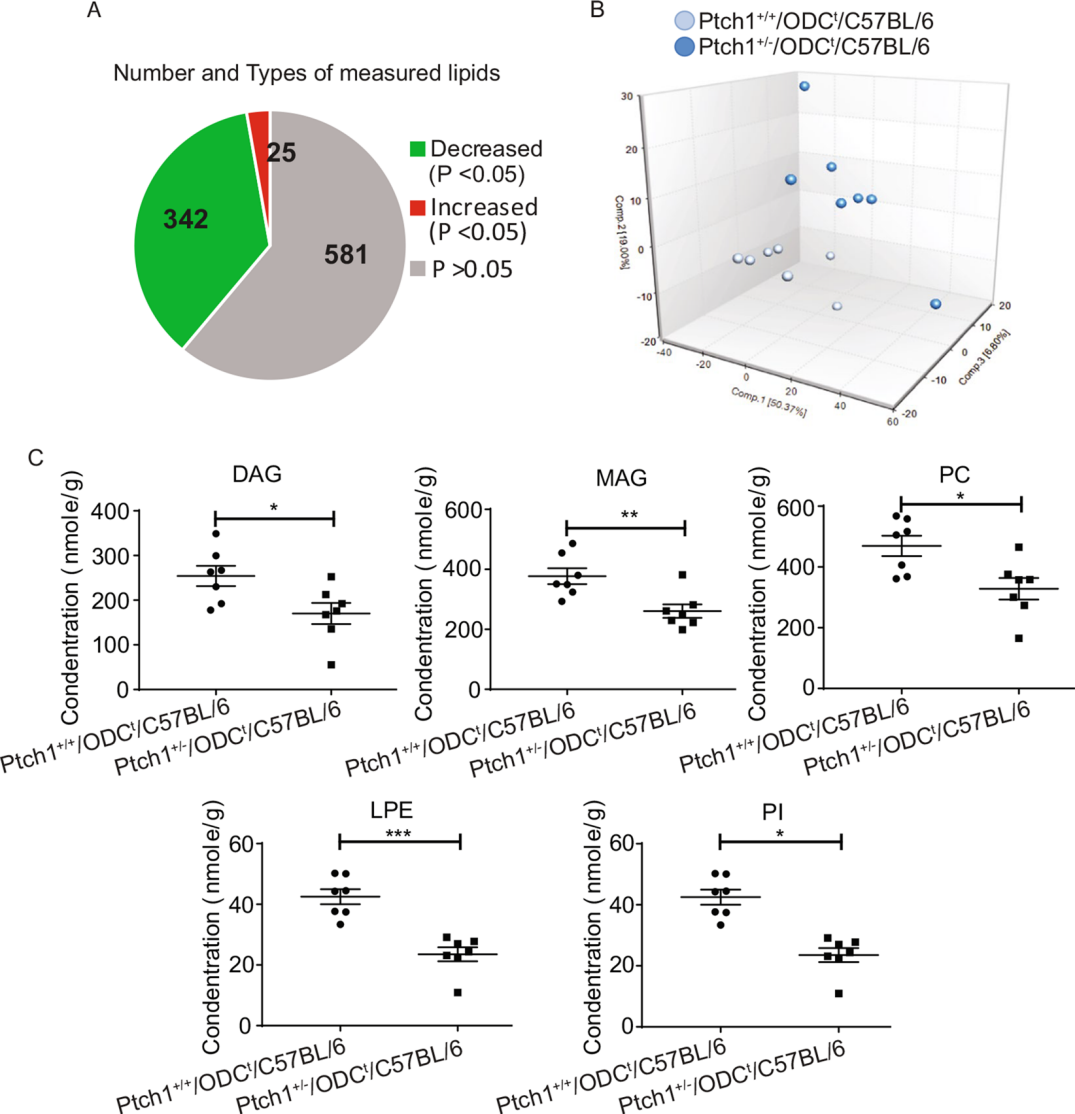
Effects of Ptch1^+/−^ heterozygosity on lipid profile in the skin. (**A**) Pie chart depicting the numbers of lipids, which are increased or decreased in the skin; (**B**) Graph showing principle component analysis of lipidomics data; (**C**) Graphs showing impact on mono & diacylglycerols, phosphatidylcholines, lysophosphatidylethanolamines, and phosphatidylinositol. *p < 0.05; **p < 0.01; ***p < 0.001.

The original Article has been corrected.

